# Measurement of Air Pollution Parameters in Montenegro Using the Ecomar System

**DOI:** 10.3390/ijerph18126565

**Published:** 2021-06-18

**Authors:** Nikola Zaric, Velibor Spalevic, Nikola Bulatovic, Nikola Pavlicevic, Branislav Dudic

**Affiliations:** 1 Faculty of Electrical Engineering, University of Montenegro, Cetinjski Put bb, 81000 Podgorica, Montenegro; zaric@ucg.ac.me (N.Z.); nbulatovic@ucg.ac.me (N.B.); 2 Department of Soils, Biotechnical Faculty, University of Montenegro, Mihala Lalaica 1, 81000 Podgorica, Montenegro; velibor.spalevic@ucg.ac.me; 3 Department of Geography, Faculty of Philosophy, University of Montenegro, D. Bojovica bb, 81400 Niksic, Montenegro; 4 ZTE Corporation, Technical Support Office in Montenegro, 81000 Podgorica, Montenegro; nikola.pavlicevic2@zte.com.cn; 5 Faculty of Management, Comenius University in Bratislava, 82005 Bratislava, Slovakia; 6 Faculty of Economics and Engineering Management, University Business Academy, 21000 Novi Sad, Serbia

**Keywords:** air pollution, air measurements, Internet of Things, sensors, air quality, PM particles

## Abstract

Particulate matter air pollution is one of the most dangerous pollutants nowadays and an indirect cause of numerous diseases. A number of these consequences could possibly be avoided if the right information about air pollution were available at a large number of locations, especially in urban areas. Unfortunately, this is not the case today. In the whole of Europe, there are just approximately 3000 automated measuring stations for PM10, and only about 1400 stations equipped for PM2.5 measurement. In order to improve this issue and provide availability of real-time data about air pollution, different low-cost sensor-based solutions are being considered both on-field and in laboratory research. In this paper, we will present the results of PM particle monitoring using a self-developed Ecomar system. Measurements are performed in two cities in Montenegro, at seven different locations during several periods. In total, three Ecomar systems were used during 1107 days of on-field measurements. Measurements performed at two locations near official automated measuring stations during 610 days justified that the Ecomar system performance is satisfying in terms of reliability and measurement precision (NRMSE 0.33 for PM10 and 0.44 for PM2.5) and very high in terms of data validity and operating stability (Ecomar 94.13%–AMS 95.63%). Additionally, five distant urban/rural locations with different traffic, green areas, and nearby industrial objects were utilized to highlight the need for more dense spatial distributions of measuring locations. To our knowledge, this is the most extensive study of low-cost sensor-based air quality measurement systems in terms of the duration of the on-field tests in the Balkan region.

## 1. Introduction

Air pollution originates from numerous sources and consists of different pollutants. One of the most dangerous air pollutants is particulate matter (PM). The sources of PM can be natural such as sea aerosol, desert or volcanic dust, fires, but more often the more toxic sources originate from human-related activities such as household heating, traffic-based fuel consumption, energy production from coal, industrial and agricultural processes [1,2,3,4,5]. PM particles are, regardless of their origin, their chemical composition, shape, and surface, very tiny and thus very harmful for the human respiratory system if inhaled, especially over a longer period of time. PM particles are mostly classified into two categories: PM10 with a diameter smaller than 10 μm, and PM2.5 with a diameter smaller than 2.5 μm. PM10 particles are mainly sufficiently large enough to remain only in the upper respiratory tract, while fine PM2.5 could easily go further throughout the respiratory tract and can infiltrate into the lower parts of the respiratory system and alveoli. In the case of ultrafine PM particles (below 0.1 μm), they may cross the endothelial barrier and enter the blood [5].

Ambient air pollution is, according to a number of reports and studies, among the four major causes of morbidity and mortality worldwide [5,6,7,8,9,10,11,12,13,14,15,16,17,18,19]. According to the Lancet Commission on pollution and health and the World Health Organization (WHO), bad air quality is responsible for between 4.2 million and 7 million deaths per year at a global level [6,7,8]. Long exposure to polluted air significantly increases the risk of other non-communicable diseases such as cardiovascular, cancer, diabetes, chronic obstructive pulmonary disease, and many others [6,20]. The WHO published that worldwide air pollutants are responsible for [6]:

- 29% of all deaths and disease from lung cancer,
- 17% of all deaths and disease from acute lower respiratory infection,
- 24% of all deaths from stroke,
- 25% of all deaths and disease from ischemic heart disease,
- 43% of all deaths and disease from chronic obstructive pulmonary disease.

Based on the European Environmental Agency (EEA) reports from 2018 to 2019 the most polluted countries in Europe are Balkan countries (Bosnia-Hercegovina, Serbia, North Macedonia, and Montenegro) and Turkey, based on both EU daily limit and EU annual limit [17,18,19]. It is important to mention that PM10 data are taken from about 3000 stations, while PM2.5 from just 1396 stations in the whole of Europe. PM10 measurements were not available from Albania, Greece, and Liechtenstein, while PM2.5 was missing from Montenegro and Serbia.

Air pollution measurements and results used in the aforementioned studies and reports are mainly based on data obtained from national automated measuring stations (AMS) that are installed and controlled by governmental authorities and agencies. These are professional and robust stations of high precision and accuracy, but their spatial distribution is very sparse, mainly due to the high costs of the equipment and the complex and expensive maintenance. In order to overcome this problem and provide more spatially frequent measurements, low-cost sensors, integrated with the Internet of Things (IoT) systems became very popular. There are a number of publications that analyze the performances of low-cost sensor-based PM measuring solutions [4,20,21,22,23,24,25,26,27,28,29,30,31,32]. It was shown that low-cost PM sensors could not replace professional stations due to their limitations in precision but could be complementarily used to enhance data availability and spatial resolution. A number of sensors were used in different laboratory and on-field measurements. One of the most extensive studies performed was presented in [20]. The following sensors were used: OPC-N2 (Alphasense), PMS5003 (Plantower), PMS7003 (Plantower) and HPMA115S0 (Honeywell). Several different sensors were also used in the research presented in [4]: SDS011 (Nova Fitness), ZH03A (Winsen), PMS7003 (Plantower), and OPC-N2 (Alphasense). In the studies [33,34], some additional sensors were tested: GP2Y (Sharp), PPD42NS (Shinyei), PMS1003 (Plantower), PSM305 (Innociple) and the SDS011 (Nova Fitness). Based on the analysis presented in [34], the sensor SDS011 has the best performance over the other sensors considered in the investigation. Additionally, according to the EU report [21], the SDS011 has sufficient reliability between 0.7 and 1. However, studies showed that the precision of the SDS011 sensor is very dependent on general meteorological data (air temperature and humidity) at deployment sites [4,20,21,22,28,29,30,33,34,35,36]. In most of the studies, it was reported that the SDS011 sensor has quite good measurement capabilities in the case of favourable meteorological conditions (moderate temperature and humidity), while in high humidity environments its precision decreases [36]. There is a similar situation regarding precision with some other low-cost sensors such as PPD42NS [35] and PMS7003 [4].

In this paper, we are presenting the results of the PM10 and PM2.5 parameters monitored with a self-developed Ecomar measuring system at several locations in Montenegro, during different time periods. According to our knowledge, this is the first study of this type in Montenegro and neighbouring countries. Measurements were performed in Podgorica at four locations during several months in 2018 and 2020, while in Pljevlja measurements were performed at three locations in 2018 and 2020. In total, three different measuring systems were used. Our intention was to examine the behavior of the Ecomar systems at different locations and environments, each of them with micro-location specifics. Namely, in most of the papers that analyze low-cost sensors, their performances are tested only near AMS. Therefore the Ecomar measuring system was tested in diverse environmental conditions at various pollution-sensitive areas. Its performances are analyzed in terms of reliability, data validity, measurement precision and operating stability.

## 2. Materials and Methods

### 2.1. Physiographic Setting

**Figure 1:**
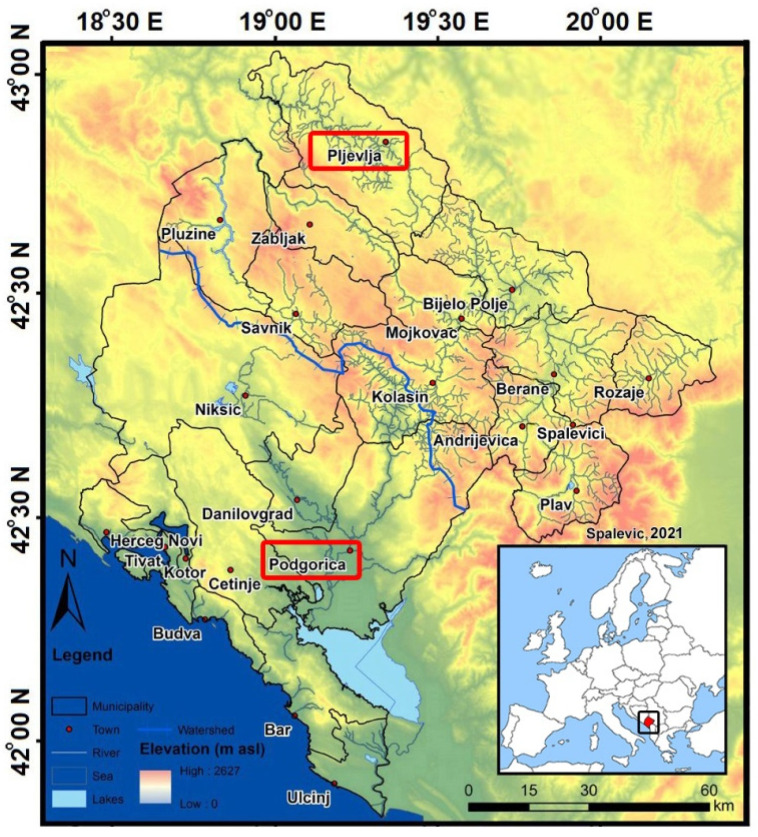
Study area with study locations of Podgorica and Pljevlja.

Montenegro lies within the Dinaric Alps, a range dominated by carbonate rocks, with some other sedimentary and volcanic strata, particularly in the north. Montenegro’s climate is strongly determined by its mountains. Besides the occurrence of important vertical temperature and rainfall gradients, local orographic rain may be important. The Dinaric Alps act as a barrier between Mediterranean and continental air masses, and this results in two major climatic zones in Montenegro. In the southern part, the Mediterranean climate prevails, whereas inland, the climate is more continental [37]. Measurements were performed in two cities in Montenegro, Podgorica and Pljevlja at seven different locations in total. Study area with study locations of Podgorica and Pljevlja is presented in the Figure 1.

Podgorica is located in the southeast part of Montenegro near Skadar Lake (largest in the Balkan region) and surrounded by high mountains on each side. The average above sea level is 49 m. The climate is characterized as the Mediterranean, with extremely warm summers (daily average 27 °C) and moderately cold winters (daily average 7.2 °C). On the other side, Pljevlja is located in north Montenegro (about 785 m above sea level), surrounded by mountains, situated in a basin. Winters are very cold and snowy with an average temperature below 0 °C, while in summer the average temperature is about 17.5 °C.

### 2.2. Measurement System Setup

The Ecomar measurement system setup, components used, and installation procedure are described in this section. Firstly, we will discuss in detail all components important for the systems integration including packaging. In the second part of this section, two typical installations of the Ecomar system are presented.

The structure of the Ecomar system is based on the following segments: control logic, sensors, communication module, and power supply. The microcontroller unit controls the data flow and data acquisition part of the system. It serves as a link between the sensor nodes, on one side, and cloud web applications on the other side. The microcontroller unit collects and handles data from multiple sensors’ measurements. For PM monitoring, particle sensor SDS011 is used, which can monitor PM2.5 and PM10 particles simultaneously [33].

The SDS011, Nova Fitness sensor, is a low-cost PM sensor, classified as an optical sensor as it measures PM10 and PM2.5 through the principle of light scattering. A small fan produces negative pressure to create a continuous airflow from the inlet to the measuring chamber. The laser beam radiates light to the air sample. A photodiode detects the amount of light dispersed by the particles, which in turn, translates the signal into electrical pulses. A microcontroller analyzes these signals and calculates the PM mass concentration based on the pulse wave amplitude. SDS011 measures concentrations from 0 to 999.9 μg/m^3^ and detects particles with a minimum diameter of 0.3 μm. The fastest sampling rate of the SDS011 sensor could be 1 sample per second, but in that case, lifespan of the sensor is very short. Considering that outdoor PM concentration is not so fast changing, a much slower sampling rate is sufficient for accurate measurement. In all the Ecomar devices, sampling is performed every 5 min, data are stored locally, while the system calculates mean value and sends this every 1 h.

Collected data are sent by the GPRS module to the web application. We selected GPRS as a unique module since Wi-Fi connection was not available at each location and the data payload is very small. The system is powered from the grid, but in some circumstances, solar panels could be an alternative solution. There is also a backup battery that enables system operation up to one day without power from the grid. Battery level is also sent, through GPRS connection, with other sensors’ measured data, so it can be tracked if there is a power shortage. The microcontroller is programmed to set the system in sleep mode whenever there is no measurement. The system is designed in a modular way so it can manage diverse sensors without important software and hardware structural changes.

Considering that the Ecomar system is intended for outdoor use, the whole data processing unit is sealed in a waterproof case with IP68 protection [38,39]. In order to provide adequate airflow through the box, two additional pipes are added, one at the bottom of the case and the other from the side. The SDS011 sensor is positioned next to the bottom pipe. Additionally the pipes are protected with mesh to provide protection from insects, leaves, large dust particles, and other dirt.

**Figure 2:**
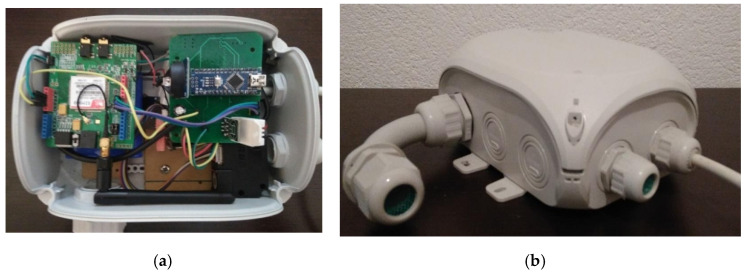
Electronics design (**a**) and IP68 air-intake enclosure (**b**) of the Ecomar system.

Our solution is based on the Arduino platform consisting of the following components (Table 1):

Electronics design and IP68 air-intake enclosure of the Ecomar measurement system is presented in the Figure 2.

### 2.3. Installation Description

**Figure 3:**
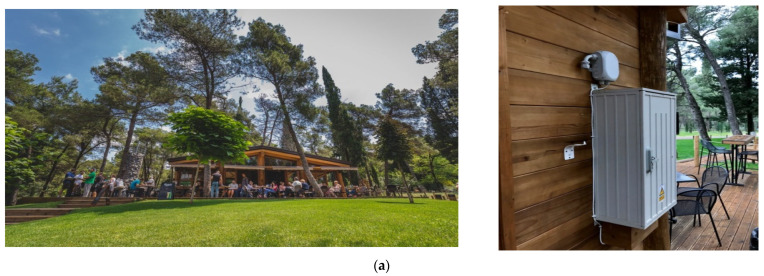

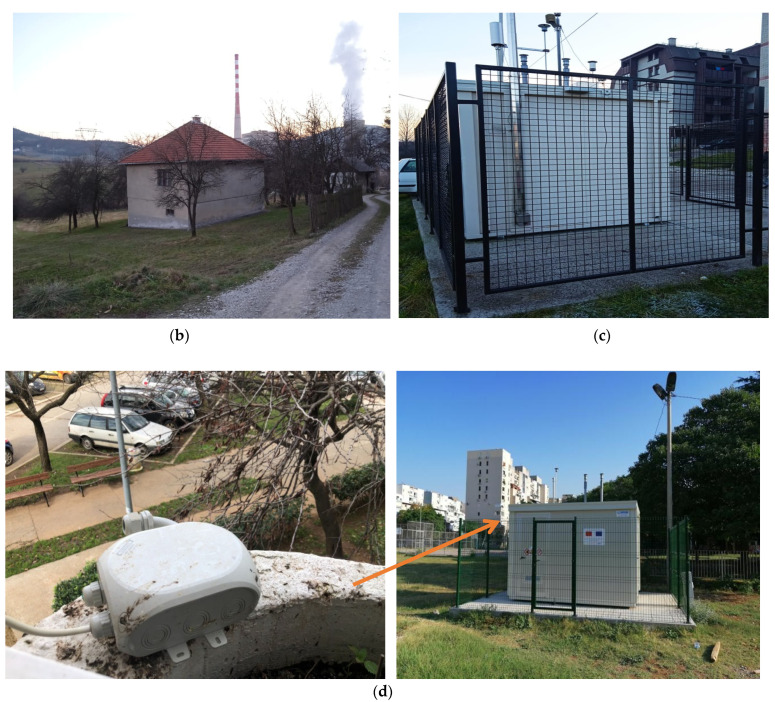
Installation setup examples: (**a**) location Podgorica 2, (**b**) location Pljevlja 2, (**c**) location Pljevlja 3, (**d**) location Podgorica 3 (Montenegro).

For the installation of the Ecomar system, several typical installation setups were used depending on the available infrastructure. One type was open space locations such as the Podgorica 2 and Pljevlja 2 locations where systems were mounted on the wall of an object at a height of 2 *m* from the ground. Objects, where the Ecomar system was installed at Podgorica 2 and Pljevlja 2, are shown in Figure 3a,b respectively. The installation setup at both locations was identical.

Other types were installations in residential buildings such as the locations of Podgorica 1, Podgorica 3 (Figure 3d), Pljevlja 1, and Pljevlja 3 (Figure 3c). For the installations in urban areas, Ecomar systems were placed on the balconies of nearby apartments at an approximate height of 3 to 4 m above the ground. We were not allowed to placed Ecomar measurement systems next to the official EPA station, thus we selected this type of setup. Additionally, due to the privacy requirements of some owners, we were not allowed to present pictures from their balconies.

## 3. Results

**Figure 4:**
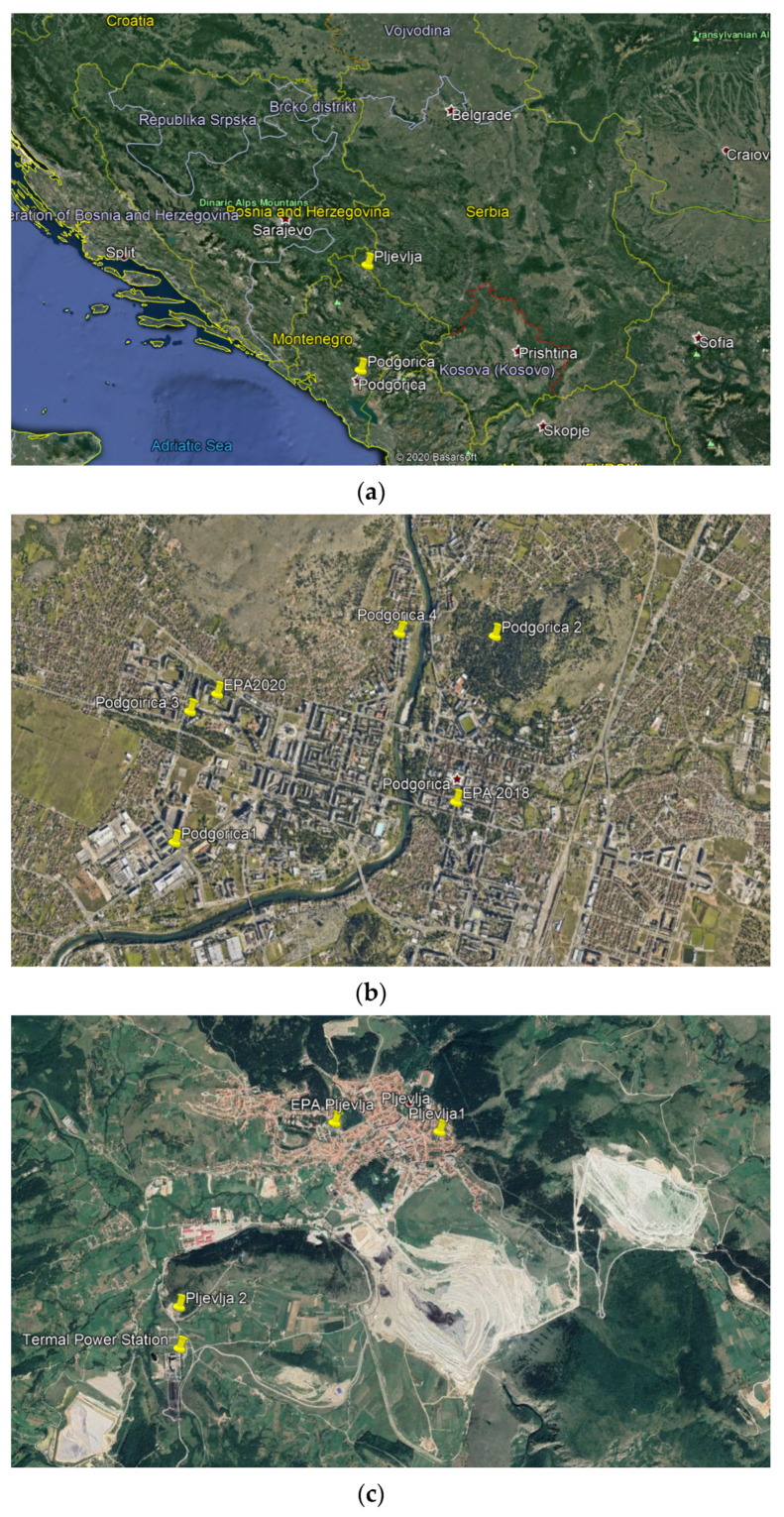
Locations of the monitoring stations: (**a**) Location of Podgorica and Pljevlja within Montenegro; (**b**) Locations of measuring sites in Podgorica; (**c**) Locations of measuring sites in Pljevlja.

In this section analysis of measurement site selection is presented as well as the data acquisition and representation process. The results relating to the precision of the Ecomar stations compared to official EPA stations are given. Furthermore, results are provided of the measurements of the Ecomar system from different spatial and characteristic locations in Podgorica and Pljevlja (Montenegro). Locations of the monitoring stations: (a) Location of Podgorica and Pljevlja within Montenegro; (b) Locations of measuring sites in Podgorica; (c) Locations of measuring sites in Pljevlja is presented in the Figure 4.

### 3.1. Measurement Site Selection

Measurements are performed at four different locations in Podgorica (Figure 2a), during 2018 at Podgorica 1 and Podgorica 2, and in 2020 at Podgorica 3 and Podgorica 4. Three different measuring sites were considered for Pljevlja (Figure 2b), in 2018 (Pljevlja 1 and Pljevlja 2) and in 2020 (Pljevlja 1 and Pljevlja 3–Pljevlja EPA). Gravimetric measuring stations of the Nature and Environmental Protection Agency Montenegro (EPA) are denoted as EPA2018 and EPA2020 in Podgorica, and EPA Pljevlja [40,41]. Note that only two locations (Podgorica 3 and Pljevlja 3) are near EPA AMS, while others are set at interesting air-pollution sites in the cities.

### 3.2. Data Acquisition and Representation

In this section, we will discuss steps in generating and preparing the PM dataset, which was obtained from three different Ecomar systems deployed at seven pollution-sensitive urban and rural Montenegrin areas.

The Ecomar system is designed to collect several PM measurements within a one-hour period. With each passing hour, measured data is averaged and the calculated result is fed into the remote database. Arrays of daily one-hour measurements are then combined and displayed as box plot diagrams.

Box plots are well suited for the explanatory visual representation of PM dataset mean value and distribution of individual measurements. Daily box plots are divided into quartile ranges which are confined by the minimum and maximum values (lowest and largest data points excluding the outliers) including the three median values (middle values of the lower half, upper half, and the whole dataset). The vertical blue rectangle, also known as the interquartile range (IQR), is calculated by subtracting the third and the first quartile values (upper and lower median values) IQR = Q3 − Q1. All data points with a distance of more than 1.5 IQR above the Q3 or below the Q1 are plotted as outliers (black circles in figures).

Besides box plots, daily mean PM values were calculated and plotted in figures. Daily mean values are used in air quality systems and sensor comparison as we were able to extract the daily mean values dataset from the EPA. Missing data points are caused by air quality sensors’ temporary malfunction. Statistical PM datasets analysis suggested we confine the PM range from 0 to 200 μg/m^3^. All the values outside the aforementioned range are supposed to be the sensors’ malfunction outliers.

### 3.3. Data Precision Comparison of Ecomar and EPA Station

**Figure 5:**
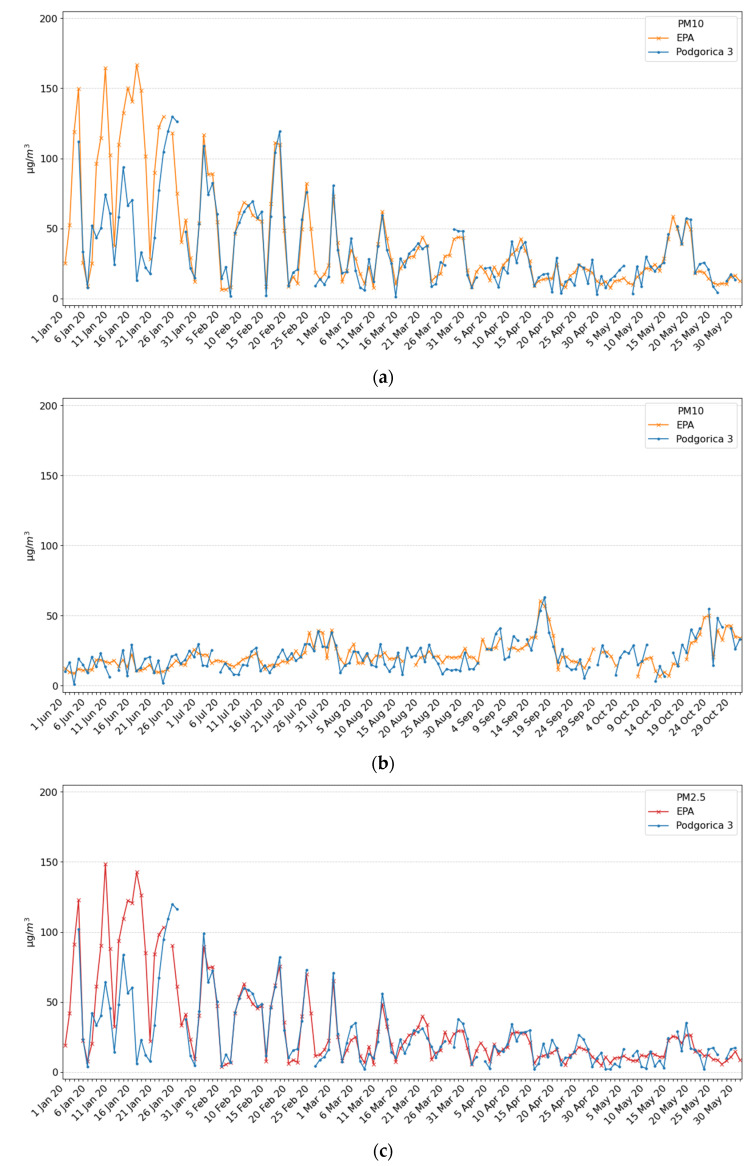

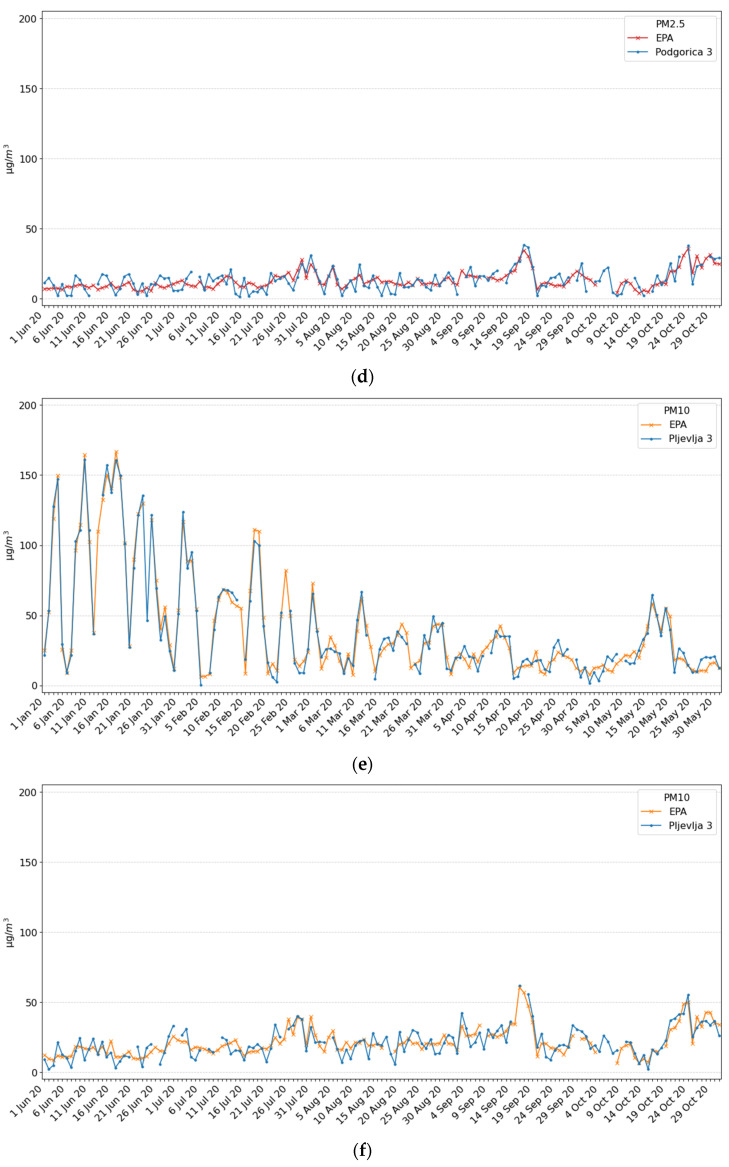

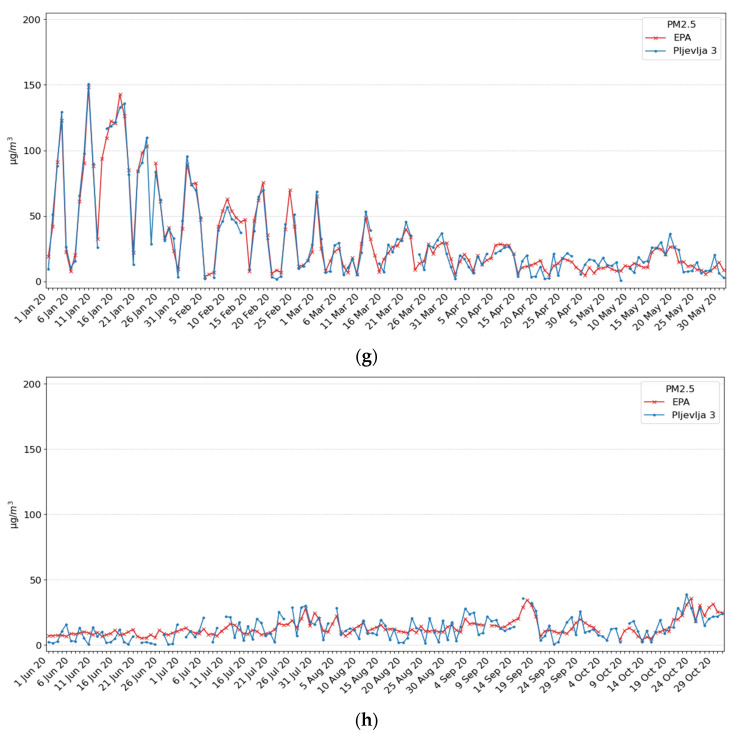
Measurement results for the Podgorica 3 and Pljevlja 3 sites and comparison with EPA stations: (**a**) PM10 particles for the Podgorica 3 location in the period January–May 2020, (**b**) PM10 particles for the Podgorica 3 location in the period June–October 2020, (**c**) PM2.5 particles for the Podgorica 3 location in the period January–May 2020, (**d**) PM2.5 particles for the Podgorica 3 location in the period June–October 2020, (**e**) PM10 particles for the Pljevlja 3 location in the period January–May 2020, (**f**) PM10 particles for the Pljevlja 3 location in the period June–October 2020, (**g**) PM2.5 particles for the Pljevlja 3 location in the period January–May 2020, (**h**) PM2.5 particles for the Pljevlja 3 location in the period June–October 2020.

After EPA Montenegro installed a new stationary station EPA2020 in the Podgorica that contains PM2.5 and PM10 measuring equipment, we installed an Ecomar device at the nearby location Podgorica 3. Ecomar measurement station Pljevlja 3 is also located close to the official EPA Montenegro station denotes EPA Pljevlja. Measurements on both locations were performed in the period from 4 January to 30 October 2020. Measurement results for the location Podgorica 3 for PM10 are shown in Figure 5a,b, while for the PM2.5 in Figure 5c,d, while for Pljevlja 3 location results are given in in Figure 5e,f for PM10 and Figure 5g,h for PM2.5. On all graphs, average daily values for the official EPA station are denoted with a red line.

Analyzing the presented result, one may observe that the average daily values for the Ecomar and EPA station were very similar both for PM10 and PM2.5, except for some periods in January for the Podgorica 3 location. In that period, after noticing the strange results and visiting the measuring site, we found that the Ecomar system was covered by a plastic bag. However, we did not omit these data in order to show what could happen during on-field measurements, and that measurement results should be checked frequently. A more detailed analysis of the result is presented in the Discussion section.

### 3.4. Spatial Analysis

**Figure 6:**
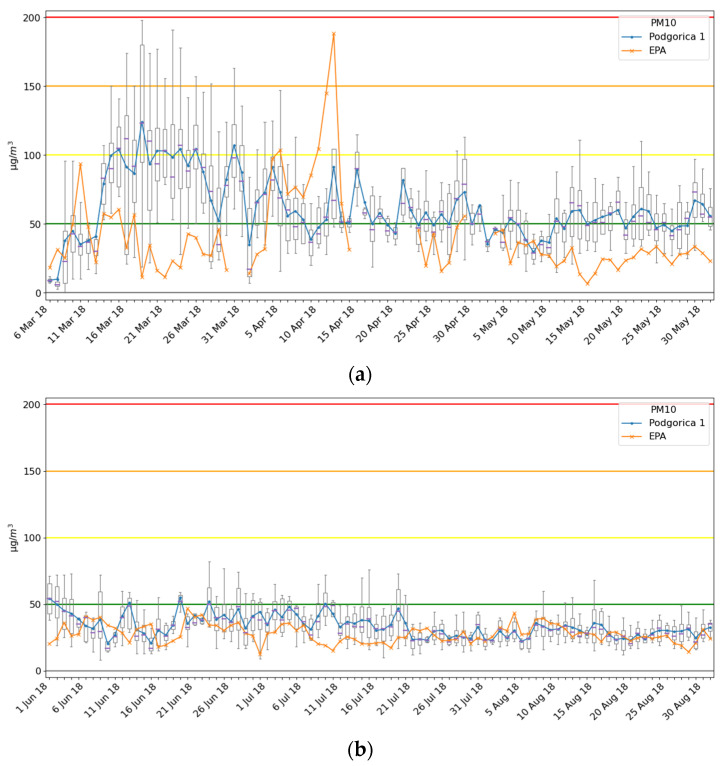
Measurement results for the Podgorica 1 site and comparison with the EPA2018 station: (**a**) period 6 March–31 May 2018; (**b**) period 1 June–31 August 2018.

Measurement data for the Podgorica 1 Ecomar system, for almost a 6 month period, are shown in Figure 6. Note that only results for PM10 are shown since in the considered period EPA Montenegro in Podgorica did not have equipment for PM2.5 measurement. Daily box plots of the Ecomar systems are denoted with blue boxes and its average daily values with the blue line, while average daily values of the EPA2018 AMS are marked with an orange line (the same notation was used in all graphics for PM10 particles). For the sake of better reading, data for the period 6 March 2018 to 31 May 2018 are shown in Figure 5a, while the period from 1 June 2018 to 31 August 2018 are reported in Figure 5b.

The system at location Podgorica 1 was installed near one of the most frequently used boulevards in the new part of the city. Additionally, at a distance lower than 300 m, there are individual houses that in the winter period mostly use wood and coal-based heating. That is the reason why average daily values of the Ecomar system during March (while the heating season is still active) are significantly higher than for the EPA2018 station, which is located in a part of the city where there are no individual houses. During that period, average daily values for both systems are quite similar, even though these two stations were at about 3 km distance. That means that pollution caused by other non-heating-related factors is similar in both parts of the city.

**Figure 7:**
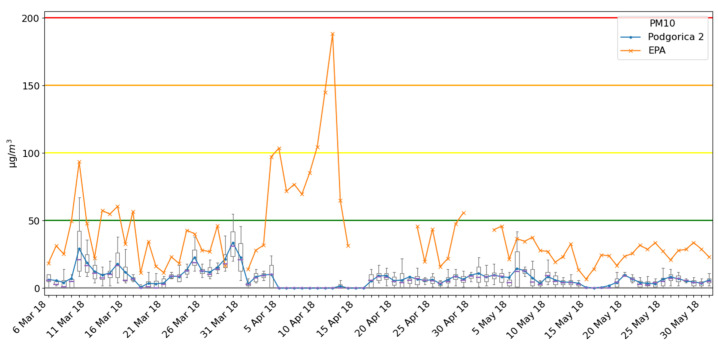
Measurement results for the Podgorica 2 site and comparison with the EPA2018 station.

Results for PM10 particles at location Podgorica 2 are presented in Figure 7, for a period of 3 months (6 March–31 May 2018). The Ecomar Podgorica 2 system was located at hill Gorica (the largest city green area) at a measuring point with a higher altitude about 40 m than one of the EPA2018 measuring points. In the area of about 500 m in radius, there are just a few residential houses and there is no traffic. Therefore, as was expected, measured values at this location were significantly lower than in the city center (location of the EPA2018 station).

**Figure 8:**
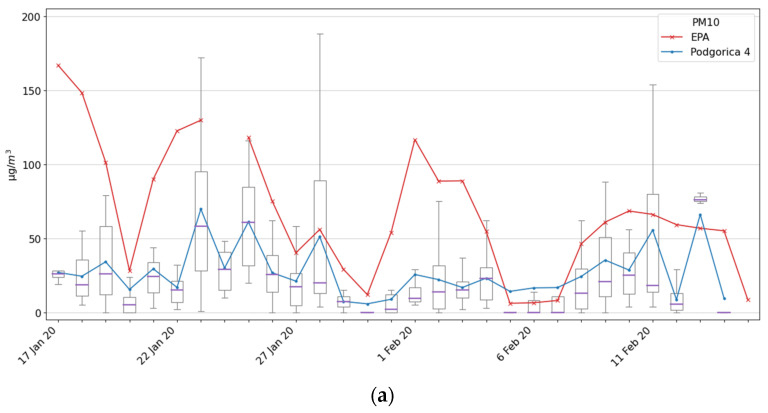

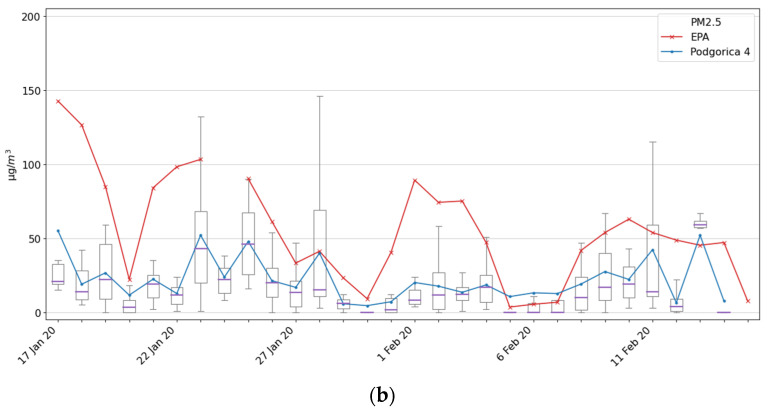
Measurement results for the Podgorica 4 site and comparison with the EPA2020 station: (**a**) PM10; (**b**) PM2.5.

The last tested location in Podgorica was Podgorica 4 location, on the 11th floor of the building near Moraca River. Why did we choose a location on the 11th floor? Namely, one of the authors of this paper lives there and he stated that air is polluted even at that height. In order to check this, we installed the Ecomar system on his terrace. Measurements were performed from 17 January to 17 February 2020, and results are shown in Figure 8a for PM10 and Figure 8b for PM2.5.

As our colleague declared, the air was polluted even on the 11th floor, with 5 days with average daily values of PM10 higher than 50 µg/m^3^ (which is the EU daily limit [18]). For the PM2.5 particles, the situation is much worse since there were 11 days with an average daily value above the EU limit of 25 µg/m^3^ and with 24 days above the WHO daily limit which is 10 µg/m^3^ [16,18]. An explanation for such high levels of both PM10 and PM2.5 might be that this location is next to the hill Malo Brdo, which has densely placed residential houses that use wood and coal-based heating.

**Figure 9:**
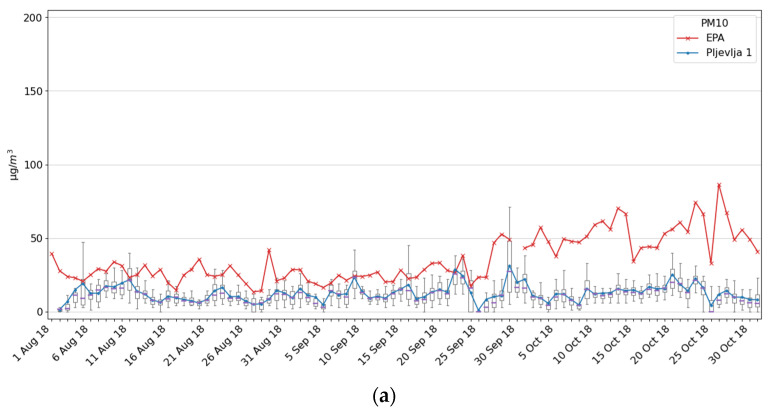

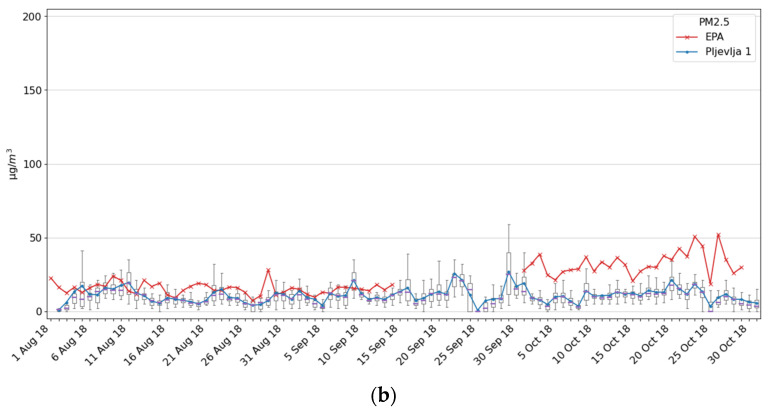
Measurement results for the Pljevlja 1 site and comparison with the EPA Pljevlja station: (**a**) PM10; (**b**) PM2.5.

The Ecomar measuring system in Pljevlja 1 was installed about 700 m from the EPA Pljevlja station during a 3 month period (1 August–30 October 2018). It is characteristic for Pljevlja that even residential and administrative buildings use wood and coal-based heating and that the heating season starts very early, sometimes at the beginning of September (Figure 9). Out of the heating season, Ecomar and EPA AMS reported similar results, while after the heating season started the Ecomar system detected slightly lower values since it was installed in the less inhabited part of the city near large green areas.

**Figure 10:**
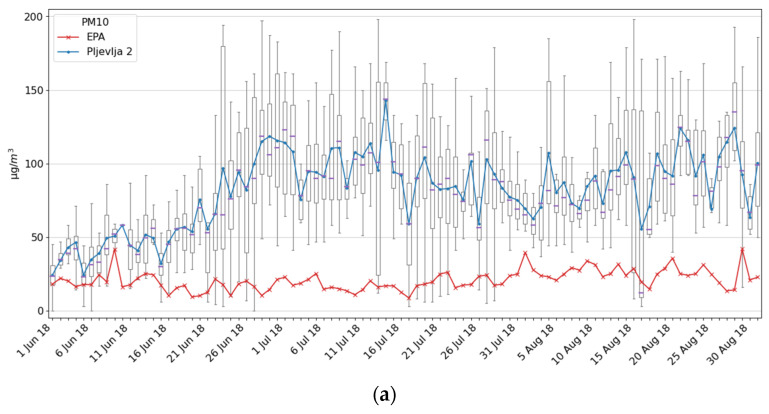

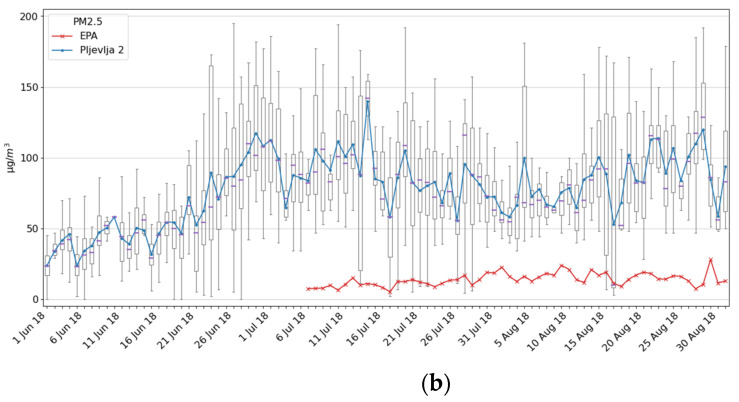
Measurement results for the Pljevlja 2 site and comparison with the EPA Pljevlja station: (**a**) PM10; (**b**) PM2.5.

Just 3 km from the Pljevlja city centre there is a Thermal Power Plant (TEP), a condensation power plant designed with two blocks of 225 MW that uses coal from a nearby mine. Thus, we installed the Pljevlja 2 station in front of the houses closest to the TEP. Intentionally we selected the summer period from 1 June until 31 August 2018. Namely, based on EPA reports for that period during previous years, pollution was not detected by the station EPA Pljevlja in the city center, while inhabitants who live near TEP complained that pollution is high even during the summer period. Unfortunately, the measurement result shown in Figure 10 confirmed citizens’ concerns about very high air pollution in that area. The PM2.5 average daily values were higher than 25 µg/m^3^ during each of the 90 days of measurement, while for the PM10 this was the case for 79 days.

## 4. Discussion

In this section results presented in the previous section are discussed in terms of comparison of the Ecomar measured data and the AMS data based on the correlation of the results, measuring site location influence, as well as operational stability of the Ecomar system.

### 4.1. Location Analysis

In total seven different locations in the two cities in Montenegro, Podgorica and Pljevlja were selected for measurements. Detailed analysis of the considered locations regarding the area in the city, traffic, green areas, and nearby industrial objects is presented in Table 2. One may observe that there are several characteristic types of locations. The first ones are urban locations near EPA stations (Podgorica 3 and Pljevlja 3). They are characterized by very intensive traffic, rare green areas, and absence of the industrial objects. These locations are primarily selected to clarify the performances of the Ecomar system in terms of measurement reliability and accuracy. All other locations are selected in order to analyze the spatial distribution of the PM particles in different and distant locations from official EPA stations. The second group is urban locations but distant from the EPA stations (Podgorica 1, Podgorica 4, and Pljevlja 1). These locations are characterized by the different intensities of the traffic, rare to medium green areas, and absence of industrial objects. The third characteristic location is Pljevlja 2 which is in a rural area with low traffic, a lot of green areas but very close to the industrial object of the thermal electric plant. The last selected location is Podgorica 2 where there is no traffic, no residential or other objects and there is a lot of trees and green areas.

### 4.2. Measurements Result Accuracy Analysis

The Ecomar performance assessment and comparison with the EPA stations is firstly performed using Normalized Root Mean Square Error (NRMSE). This was calculated with daily mean PM10 and PM2.5 parameters (Table 3). The Ecomar system daily mean PM measurements (denoted as a_i_) are compared to referent EPA stations daily mean PM measurements (denoted as b_i_).

(1) $$\mathrm{NRMSE}=\frac{\sqrt{\frac{1}{N}\sum_{i=1}^{N}{{\left(a_{i}-b_{i}\right)}^{2}}^{}}}{\frac{1}{2N}\sum_{i=1}^{N}{\left(a_{i}-b_{i}\right)}^{2}}$$

In order to further investigate performances of the Ecomar system relating to data validity, the PM10 and PM2.5 measurements are analyzed in terms of maximal allowed average daily values, which are according to Montenegrin and EU limits set to 50 µg/m^3^ for PM10 and 25 µg/m^3^ for PM2.5. The results for the Ecomar and EPA AMS are given in Table 4. Note that the number of days where the Ecomar readings are above the daily limit are quite similar to those of the EPA stations.

Analyzing the overall results given in Table 3 and Table 4 one may conclude that the Ecomar stations produce sufficiently accurate results compared to the EPA Montenegro stations. As such they could be qualified as an appropriate supplemental station suitable for large-scale deployment that will enable widespread measurement and better spatial resolution of air quality regarding PM particle concentration. Note that results for stations in the period January–May are somewhat unfavourable, but this is a consequence of physical influence on the Ecomar system. As already mentioned in the previous section, we found that the Ecomar system at location Podgorica 3 was covered with a plastic bag for some period. If we omit this period from the calculations, results will be quite similar to all others.

Note that presented results are obtained directly as sensor readings without taking into account other parameters such as temperature and relative humidity. Taking into account these parameters as corrective ones, more precise results of the SDS011 sensor measurements could be obtained, as suggested in [4]. However, these corrections are very dependent on exact meteorological data, location and particle structure as stated in [36].

Therefore, more extensive analysis of the temperature and humidity influence for the Mediterranean region, of which Montenegro belongs, should be performed prior to large scale deployment of any type of low-cost based measuring devices. Additionally, it is important to define confidence intervals of the sensor readings in order to avoid false-positive or false-negative results.

### 4.3. Spatial Analysis

In order to highlight the importance of the existence of a more dense spatial distribution of the measuring stations, five quite different locations were considered. Since it is not suitable to compare the Ecomar and EPA stations in terms of NRMSE for these locations, we only present results for PM10 and PM2.5 measurements in terms of maximal allowed average daily values. The results for the Ecomar and EPA stations are given in Table 5.

Note that the numbers of the Ecomar readings that are above the daily limit are only higher than corresponding ones for the EPA stations for the Podgorica 1 and Pljevlja 2 locations. For Podgorica 1, this is a consequence of readings during March 2018 being during the heating season at that location, while during the rest of the measurement period, readings are very similar. Results for Pljevlja 2 indicate that even during the summer period when pollution is expected to be lower, based on the readings from the EPA station located in the city, we had readings that were extremely high, which is a consequence of the TEP proximity. Even though temperature and humidity data were not available during this experiment, the data might be considered as sufficiently precise since measurements at the Pljevlja 2 location were performed during the summer period when relative humidity is quite low.

### 4.4. Operational Stability

One of the most important characteristics of any measuring device, except precision, is operational stability. Operational stability means that data obtained during a 24-h measurement are valid for analysis. Measurements during one day are considered valid if more than 75% of hourly readings were available [18]. Thus we analyzed performances of the Ecomar systems in terms of the number of days when there were not valid measurements, i.e., when we were not able to calculate average daily values. The results of operational stability analysis for the Ecomar and EPA systems are presented in Table 6.

For the Ecomar system in total, there were 1107 days of measurements for three deployed systems at seven different locations. One Ecomar system was used for Podgorica 1 and Podgorica 4 sites (223 days), second in Podgorica 2, Pljevlja 1 and Pljevlja 3 (487 days), and third one in Podgorica 3 and Pljevlja 2 (397 days). In total there were only 65 days when the Ecomar system was out of order or data were not valid for analysis, which is just 5.87% of the total 1107 days when measurements were performed. For the EPA stations PM10 readings, statistics are somewhat better (4.37%) than the Ecomar, while for the EPA PM2.5 the sensor was not working for 66 out of 838 days (7.87%) when it was installed.

Thus, one may conclude that the operational stability of the Ecomar system is very high, especially bearing in mind that measurements are performed using three systems at seven different locations during all seasons. Additionally, according to our knowledge, this study has reported on the longest testing period and has the largest number of locations.

## 5. Conclusions

In this study, we presented on-field results of PM particle measurements using a self-developed Ecomar system that integrates a low-cost SDS011 sensor. Measurements were performed in two cities in Montenegro, at seven different locations for 1107 days in total. According to our knowledge, this study has the longest testing period at the largest number of locations in the Balkan region, which is one of the most polluted European regions.

Based on the results, we can conclude that measurements of the low-cost sensor-based systems are not as accurate as for AMS, but they could provide a sufficiently precise estimation of the PM level with higher spatial and temporal resolution. This is especially noticeable for the field tests performed in Pljevlja, near the thermal power station, during the summer period. AMS located in the city did not register any measurement that exceeded the average daily limit, while our station located near the thermal power station registered very high values during the whole test period. Additionally, measurements in Podgorica showed that micro-location characteristics significantly influence the air quality and that one AMS for the whole area of the Podgorica city is not sufficient to adequately represent air pollution levels.

Additionally, we proved that the operational stability of the Ecomar system is very high, especially bearing in mind that measurements were performed by using three systems at seven different locations during 1107 days within all seasons.

Finally, we may conclude that low-cost sensor-based solutions, such as Ecomar, could significantly improve temporal and spatial availability of air pollution data, which could be of high importance for human health-risk assessments. Future research will be oriented toward the improvement of system accuracy as well as the development of a notification and recommendation system that will warn citizens to adjust physical activities in the case of highly-polluted air. Additionally, special attention in future research will be given to the analysis of temperature and humidity influence to data precision, as well as identification of confidence intervals of data validity.

## Figures and Tables

**Table 1:** Components of the Ecomar system.

Electronic Components
Arduino Nano 3.0 microcontroller board
Printed Circuit Board
GSM/GPRS SIM900 shield module
SDS011 Particulate Matter sensor
DS3231 Real Time Clock module
Lithium Ion Battery 6600 mAh
Solar power module (max power 3 W, max power voltage 5.8 V, max power current 520 mA)
IP68 waterproof enclosure with custom designed air-intake component

**Table 2:** Measurement locations analysis.

Location	EPA Station Distance	Area in the City	Traffic	Green Areas	Industrial Object
Podgorica 1	3 km	Urban	Very intensive	Rare	No
Podgorica 2	1.5 km	Not populated within 500 m	No	Dense (largest green area in the City)	No
Podgorica 3	20 m	Urban	Very intensive	Medium (mostly grass)	No
Podgorica 4	3 km	Urban	Medium	Low	No
Pljevlja 1	700 km	Urban	Medium	Nearby large green areas	No
Pljevlja 2	3 km	Rural	Rare rural traffic	Dense	Yes
Pljevlja 3	30 m	Urban	Intensive	Rare	No

**Table 3:** NRMSE analysis of the daily mean PM10 and PM2.5 values measured by the Ecomar systems and referent EPA stations in 2020.

System Label	NRMSE	
	PM10	PM2.5
Podgorica 3 ^Jan–May 2020^	0.6705	0.7562
Podgorica 3 ^Jun–Oct 2020^	0.2953	0.4019
Pljevlja 3 ^Jan–May 2020^	0.1375	0.1725
Pljevlja 3 ^Jun–Oct 2020^	0.2661	0.4761

**Table 4:** Daily mean PM10 and PM2.5 values of the Ecomar and referent EPA stations related to allowed daily limits.

System Label	Average Daily Limit PM10 > 50 µg/m^3^		Average Daily Limit PM2.5 > 25 µg/m^3^	
	Ecomar	EPA	Ecomar	EPA
Podgorica 3 ^Jan–May 2020^	35 of 152	36 of 152	53 of 152	58 of 152
Podgorica 3 ^Jun–Oct 2020^	3 of 153	2 of 153	11 of 153	8 of 153
Pljevlja 3 ^Jan–May 2020^	38 of 152	36 of 152	63 of 152	59 of 152
Pljevlja 3 ^Jun–Oct 2020^	3 of 153	1 of 153	14 of 153	9 of 153

**Table 5:** Daily mean PM10 and PM2.5 values of the Ecomar and referent EPA stations related to allowed daily limits.

System Label	Average Daily Limit PM10 > 50 µg/m^3^		Average Daily Limit PM2.5 > 25 µg/m^3^	
	Ecomar	EPA	Ecomar	EPA
Podgorica 1 ^Mar–Aug 2018^	51 of 179	16 of 179	156 of 179	NA ^1^
Podgorica 2 ^Mar–May 2018^	0 of 90	16 of 90	2 of 90	NA ^1^
Podgorica 4 ^Jan–Feb 2020^	5 of 44	20 of 44	8 of 44	22 of 44
Pljevlja 1 ^Aug–Oct 2018^	0 of 92	17 of 92	2 of 92	26 of 92
Pljevlja 2 ^Jun–Aug 2018^	79 of 92	0 of 92	90 of 92	1 of 92

^1^ EPA data were not available.

**Table 6:** Operation stability analysis for the Ecomar and referent EPA stations.

System Label	Ecomar		EPA PM10		EPA PM2.5	
	Number of Days	%	Number	%	Number	%
Podgorica 1 ^Mar–Aug 2018^	0 of 179	0	13 of 179	7.26	NA ^1^	NA ^1^
Podgorica 2 ^Mar–May 2018^	21 of 90	23.3	13 of 90	14.4	NA ^1^	NA ^1^
Podgorica 3 ^Jan–May 2020^	11 of 152	7.24	1 of 152	0.66	1 of 152	0.66
Podgorica 3 ^Jun–Oct 2020^	10 of 153	6.54	9 of 153	5.88	5 of 153	3.27
Podgorica 4 ^Jan–Feb 2020^	3 of 44	6.8	1 of 44	2.3	1 of 44	2.3
Pljevlja 1 ^Aug–Oct 2018^	1 of 92	1.1	1 of 92	1.1	18 of 92	19.5
Pljevlja 2 ^Jun–Aug 2018^	0 of 92	0	0 of 92	0	35 of 92	38.8
Pljevlja 3 ^Jan–May 2020^	9 of 152	5.92	1 of 152	0.66	1 of 152	0.66
Pljevlja 3 ^Jun–Oct 2020^	10 of 153	6.54	9 of 153	5.88	5 of 153	3.27
Total	65 of 1107	5.87	48 of 1107	4.37	66 of 838	7.87

^1^ EPA data were not available.

## References

[1] Anderson J.O., Thundiyil J.G., Stolbach A. (2012). Clearing the air: A review of the effects of particulate matter air pollution on human health. J. Med. Toxicol..

[2] Kim K.H., Kabir E., Kabir S. (2015). A review on the human health impact of airborne particulate matter. Environ. Int..

[3] Guarnieri M., Balmes J.R. (2014). Outdoor air pollution and asthma. Lancet.

[4] Badura M., Batog P., Drzeniecka-Osiadacz A., Modzel P. (2018). Evaluation of Low-Cost Sensors for Ambient PM2.5 Monitoring. J. Sens..

[5] IQAIR (2020). 2019 World Air Quality Report Region & City PM2.5 Ranking. IQAir.

[6] WHO https://www.who.int/airpollution/ambient/health-impacts/en/.

[7] Landrigan P.J., Fuller R., Acosta N.J., Adeyi O., Arnold R., Baldé A.B., Bertollini R., Bose-O’Reilly S., Boufford J.I., Breysse P.N. (2018). The Lancet Commission on pollution and health. Lancet.

[8] Stanaway J.D., Afshin A., Gakidou E., GBD 2017 Risk Factor Collaborators (2018). Global, regional, and national comparative risk assessment of 84 behavioral, environmental and occupational, and metabolic risks or clusters of risks for 195 countries and territories, 1990–2017: A systematic analysis for the global burden of disease study 2017. Lancet.

[9] Kerschhofer A., Breitegger P., Bergmann A. (2018). Laser Driver and Analysis Circuitry Development for Quartz-Enhanced Photoacoustic Spectroscopy of NO_2_ for IoT Purpose. Proceedings.

[10] Benka-Coker M.L., Clark M.L., Rajkumar S., Young B.N., Bachand A.M., Balmes J.R., Brook R., Nelson T.L., Volckens J., Reynolds S.J. (2018). Exposure to Household Air Pollution from Biomass Cookstoves and Levels of Fractional Exhaled Nitric Oxide (FeNO) among Honduran Women. Int. J. Environ. Res. Public Health.

[11] Qiu H., Tan K., Long F., Wang L., Yu H., Deng R., Long H., Zhang Y., Pan J. (2018). The Burden of COPD Morbidity Attributable to the Interaction between Ambient Air Pollution and Temperature in Chengdu, China. Int. J. Environ. Res. Public Health.

[12] Devakumar D., Qureshi Z., Mannell J., Baruwal M., Sharma N., Rehfuess E., Saville N.M., Manandhar D.S., Osrin D. (2018). Women’s Ideas about the Health Effects of Household Air Pollution, Developed through Focus Group Discussions and Artwork in Southern Nepal. Int. J. Environ. Res. Public Health.

[13] Cohen A.J., Brauer M., Burnett R., Anderson H.R., Frostad J., Estep K., Balakrishnan K., Brunekreef B., Dandona L., Dandona R. (2017). Estimates and 25-year trends of the global burden of disease attributable to ambient air pollution: An analysis of data from the Global Burden of Diseases Study 2015. Lancet.

[14] Brauer M., Freedman G., Frostad J., Van Donkelaar A., Martin R.V., Dentener F., Dingenen R.V., Estep K., Amini H., Apte J.S. (2016). Ambient Air Pollution Exposure Estimation for the Global Burden of Disease 2013. Environ. Sci. Technol..

[15] Lelieveld J., Klingmüller K., Pozzer A., Pöschl U., Fnais M., Daiber A., Münzel T. (2019). Cardiovascular disease burden from ambient air pollution in Europe reassessed using novel hazard ratio functions. Eur. Heart J..

[16] World Health Organization (2016). Ambient Air Pollution: A Global Assessment of Exposure and Burden of Disease.

[17] European Environmental Agency (2018). Air Quality in Europe—2018 Report.

[18] European Environmental Agency (2019). Air Quality in Europe—2019 Report.

[19] Colovic Daul M., Kryzanowski M., Kujundzic O. (2019). Air Pollution and Human Health: The Case of the Western Balkans. UN Environ..

[20] Bulot F.M.J., Johnston S.J., Basford P.J., Easton N.H.C., Apetroaie-Cristea M., Foster G.L., Morris A.K.R., Cox S.J., Loxham M. (2019). Long-term field comparison of multiple low-cost particulate matter sensors in an outdoor urban environment. Sci. Rep..

[21] Karagulian F., Gerboles M., Barbiere M., Kotsev A., Lagler F., Borowiak A. (2019). Review of Sensors for Air Quality Monitoring, EUR 29826 EN.

[22] Tagle M., Rojas F., Reyes F., Vásquez Y., Hallgren F., Lindén J., Kolev D., Watne Å.K., Oyola P. (2020). Field performance of a low-cost sensor in the monitoring of particulate matter in Santiago, Chile. Environ. Monit. Assess.

[23] Jovašević-Stojanović M., Bartonova A., Topalović D., Lazović I., Pokrić B., Ristovski Z. (2015). On the use of small and cheaper sensors and devices for indicative citizen-based monitoring of respirable particulate matter. Environ. Pollut..

[24] Trilles S., Vicente A.B., Juan P., Ramos F., Meseguer S., Serra L. (2019). Reliability Validation of a Low-Cost Particulate Matter IoT Sensor in Indoor and Outdoor Environments Using a Reference Sampler. Sustainability.

[25] Zikova N., Masiol M., Chalupa D.C., Rich D.Q., Ferro A.R., Hopke P.K. (2017). Estimating hourly concentrations of PM2.5 across a metropolitan area using low-cost particle monitors. Sensors.

[26] Johnston S.J., Basford P.J., Bulot F.M.J., Apetroaie-Cristea M., Easton N.H.C., Davenport C., Foster G.L., Loxham M., Morris A.K.R., Cox S.J. (2019). City Scale Particulate Matter Monitoring Using LoRaWAN Based Air Quality IoT Devices. Sensors.

[27] Kang J., Hwang K.-I. (2016). A Comprehensive Real-Time Indoor Air-Quality Level Indicator. Sustainability.

[28] Karagulian F., Barbiere M., Kotsev A., Spinelle L., Gerboles M., Lagler F., Redon N., Crunaire S., Borowiak A. (2019). Review of the Performance of Low-Cost Sensors for Air Quality Monitoring. Atmosphere.

[29] Budde M., Müller T., Laquai B., Streibl N., Schwarz A., Schindler G., Riedel T., Beigl M., Dittler A. Suitability of the Low-Cost SDS011 Particle Sensor for Urban PM-Monitoring. Proceedings of the 3rd International Conference on Atmospheric.

[30] Budde M., Schwarz A.D., Müller T., Laquai B., Streibl N., Schindler G., Köpke M., Riedel T., Dittler A., Beigl M. (2018). Potential and Limitations of the Low-Cost SDS011 Particle Sensor for Monitoring Urban Air Quality. ProScience.

[31] Zheng T., Bergin M.H., Johnson K.K., Tripathi S.N., Shirodkar S., Landis M.S., Sutaria R., Carlson D.E. (2018). Field evaluation of low-cost particulate matter sensors in high- and low-concentration environments. Atmos. Meas. Tech..

[32] Johnson K.K., Bergin M.H., Russell A.G., Hagler G.S. (2018). Field Test of Several Low-Cost Particulate Matter Sensors in High and Low Concentration Urban Environments. Aerosol Air Qual. Res..

[33] Jayaratne R., Liu X., Thai P., Dunbabin M., Morawska L. (2018). The influence of humidity on the performance of a low-cost air particle mass sensor and the effect of atmospheric fog. Atmos. Meas. Tech..

[34] Jayaratne R., Liu X., Ahn K.H., Asumadu-Sakyi A., Fisher G., Gao J., Mabon A., Mazaheri M., Mullins B., Nyaku M. (2020). Low-cost PM2.5 Sensors: An Assessment of their Suitability for Various Applications. Aerosol Air Qual. Res..

[35] Bai L., Huang L., Wang Z., Ying Q., Zheng J., Shi X., Hu J. (2020). Long-term Field Evaluation of Low-cost Particulate Matter Sensors in Nanjing. Aerosol Air Qual. Res..

[36] Liu H.-Y., Schneider P., Haugen R., Vogt M. (2019). Performance Assessment of a Low-Cost PM2.5 Sensor for a near Four-Month Period in Oslo, Norway. Atmosphere.

[37] Nyssen J., Van Den Branden J., Spalevic V., Frankl A., Van De Velde L., Curovic M., Billi P. (2014). Twentieth century land resilience in Montenegro and consequent hydrological response. Land Degrad Dev..

[38] Nova SDS011. http://www.inovafitness.com/en/a/chanpinzhongxin/95.html.

[39] Hensel Electric www.hensel-electric.eu/en/produkte/index-relaunch.php?IdTreeGroup=7529&IdProduct=11064.

[40] EPA https://epa.org.me/wp-content/uploads/2019/11/Septembar-2019-ok.pdf.

[41] Akreditacija http://www.akreditacija.me/SERTIFIKATI/Laboratorije_za_ispitivanje/dodatak%20Sertifikatu%20CETI%20od%2023.04.2018.pdf.

